# Up-dosing with bilastine results in improved effectiveness in cold contact urticaria

**DOI:** 10.1111/all.12171

**Published:** 2013-06-06

**Authors:** K Krause, A Spohr, T Zuberbier, M K Church, M Maurer

**Affiliations:** Department of Dermatology and Allergy, Allergie-Centrum-Charité, Charité – Universitätsmedizin BerlinBerlin, Germany

**Keywords:** bilastine, cold contact urticaria, cytokines, H_1_-antihistamine, histamine

## Abstract

**Background:**

Cold contact urticaria (CCU) is characterized by itchy wheal and flare responses due to the release of histamine and other pro-inflammatory mediators after exposure to cold. The treatment of choice is nonsedating antihistamines, dosages of which may be increased up to fourfold if standard doses are ineffective. Here, we assess the effects of a standard 20 mg dose and up-dosing to 40 and 80 mg of bilastine in reducing the symptoms of CCU and inflammatory mediator release following cold challenge.

**Methods:**

Twenty patients with CCU were included in this randomized, crossover, double-blind, placebo-controlled 12-week study. They received placebo, 20, 40 or 80 mg of bilastine daily each for 7 days with 14-day washout periods. The primary readout was change in critical temperature thresholds (CTT). Secondary readouts were changes in pruritus, levels of histamine IL-6, IL-8 and TNF-α collected by skin microdialysis and safety and tolerability of bilastine.

**Results:**

Bilastine 20 mg was highly effective (*P* < 0.0001) in reducing CTT. Up-dosing to 80 mg significantly (*P* < 0.04) increased its effectiveness. At this dose, 19 of 20 (95%) patients responded to treatment, with 12 of 20 (60%) becoming symptom free. Only one patient was refractory to treatment. Microdialysis levels of histamine, IL-6 and IL-8 assessed 1–3 h after cold challenge were significantly (*P* < 0.05) decreased following up-dosing with 80 mg bilastine. Bilastine treat-ment was well tolerated without evidence of increased sedation with dose escala-tion.

**Conclusions:**

Bilastine was effective in reducing the symptoms of patients with CCU. Increased efficacy of bilastine with fourfold up-dosing was without sedation and supports urticaria treatment guidelines.

The current EAACI/GA^2^LEN/EDF/WAO guidelines for the treatment for urticaria 1 recommend first-line use of new-generation, nonsedating H_1_-antihistamines and provide an option to increase the dosage up to fourfold if standard doses are not effective. In this study, we tested the validity of this recommendation by comparing the effectiveness of the H_1_-antihistamine, bilastine, at the manufacturer's recommended standard dose with up-dosing to four times the standard dose.

Bilastine is a new H_1_-antihistamine approved recently for the symptomatic treatment for allergic rhinoconjunctivitis and urticaria. *In vitro* studies 2 have shown bilastine to have moderate-to-high affinity and potent activity at the histamine H_1_-receptor while having negligible affinity or effects at 30 other receptors. A review of preliminary clinical data 3 has shown that bilastine is rapidly and effectively absorbed, undergoes negligible metabolism 4 and is a substrate for P-glycoprotein 5, which limits its passage across the blood–brain barrier. At the recommended dose of 20 mg, bilastine is nonsedative, does not enhance the effects of alcohol or lorazepam, does not impair actual driving tests 6 and shows no cardiotoxicity 7, 8. Bilastine's long duration of action, efficacy and freedom from central nervous system sedative effects make it a potentially attractive option for use in this clinical situation.

The model used to study bilastine was cold contact urticaria (CCU), a rare but severe and potentially lethal condition. It is characterized by itchy wheal and flare responses due to the release of histamine and other pro-inflammatory mast cell mediators such as tumour necrosis factor-α (TNF-α), prostaglandin D_2_ (PGD_2_) and platelet-activating factor (PAF) following exposure of the skin to the cold 9. Life-threatening angioedema and anaphylaxis may occur after ingestion of cold foods and systemic exposure to cold, respectively 9, 10. Because of its potential severity, it is essential that CCU is treated with the most effective therapeutic agents. In addition to the recommended standard treatment with second-generation antihistamines, the use of leukotriene antagonists, ciclosporin, antibiotics and anti-IgE therapy has been reported for therapy-refractive cases 1.

Recent data have shown that updosing of antihistamines is significantly more effective in reducing symptoms in CCU than standard-dose treatment 11, 12. However, there is still a need for identifying the effects of high-dose antihistamines compared with standard doses on mast cell mediator release, such as histamine and cytokines, in urticaria patients.

It has recently become possible to assess the severity of cold contact urticaria by measuring critical temperature thresholds (CTT) for the production of wheal responses on discrete areas of the skin using a Peltier effect-based electronic device 9, 12–14. This method has been previously compared with the ice cube challenge tests and found to be more suitable for performing standardized threshold testing 13.

With this study, we aimed at evaluating dose-dependent antihistaminic effects of bilastine on symptom development (e.g. temperature thresholds) and mast cell mediator release in CCU. The primary objective of the present cold urticaria provocation study was to assess changes in CTT for production of wheal responses in the skin following treatment with bilastine at 20, 40 and 80 mg daily for seven days. The secondary objectives were to evaluate the safety and tolerability and to use dermal microdialysis to assess the potential anti-inflammatory effects of bilastine in reducing histamine and cytokine release following cold provocation.

## Methods

**Figure 1:**
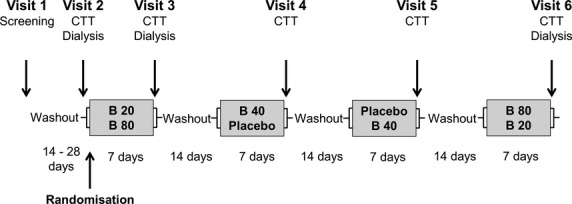
Study design: This was a double-blind, placebo-controlled, triple cross-over study of 20 patients with cold contact urticaria (CCU) treated daily for 7 days with bilastine (B) 20, 40 and 80 mg and placebo. Measurements were taken of critical temperature threshold (CTT) and skin microdialysis performed to assess histamine and cytokine generation.

This was a prospective, randomized, double-blind, placebo-controlled, triple cross-over study to assess the efficacy, mechanisms and safety of treatment with 20 mg, 40 mg, 80 mg of bilastine and placebo administered once daily for 7 days in patients with cold contact urticaria. The design of the study is shown in Fig. 1. A total of 20 patients (seven men and 13 women, mean age 45 years, range 22–68 years) with a confirmed diagnosis of CCU made at least 6 weeks before screening were included in this study. The group size was estimated using a power of 80% (*t*-test) with a two-sided significance level of 5% and a medium effect of 1.2 SD. The patients were drawn from the outpatient population at the urticaria specialty clinic of the Allergie-Centrum-Charité-Universitätsmedizin and associated dermatological institutions in Berlin, Germany.

The study was approved by the ethics committee of the State of Berlin (EudraCT number: 2010-019344-39) and was conducted according to the Declaration of Helsinki and the ‘Good Clinical Practice, Standard Operating Procedures of the Allergy Centre Charité and the Coordination Centre for Clinical Studies of the Charité-Universitätsmedizin Berlin’. Its clinicaltrials.gov identifier number is NCT 01271075. Recruitment began in December 2010 and the study completed in August 2011. All participants gave signed informed consent at the beginning of the study.

### Study design

At the screening visit, information about concomitant disease and previous medication use was collected and the patients were subjected to a general physical examination, laboratory blood analyses and an electrocardiogram. A cold provocation test was performed, and patients who exhibited a positive reaction comprising wheal and itching within 10 min of cold provocation with Temp Test 3.0® (emoSystems, Berlin, Germany) at 4°C were eligible for the study provided they were not excluded for other reasons.

The exclusion criteria were a documented or suspected history of allergic disease; any acute or chronic disease; symptoms of a clinically significant illness, especially liver or kidney disease; a history of hypersensitivity to the study drug(s) or formulation ingredients; epilepsy or other seizure conditions; hereditary galactose intolerance; lactase deficiency or glucose-galactose malabsorption; drug abuse or excessive use of alcohol or tobacco. Pregnant or nursing women were also excluded. Oral antihistamines, antidepressants, antipsychotics or corticosteroids, aluminium- and magnesium-containing antacids, ketoconazole and erythromycin, as well as topically applied antihistamines, corticosteroids or mast cell stabilizers were forbidden for 2 weeks prior to testing. Further, participants were forbidden to consume citrus fruits for 24 h before or during study days due to their possible effects on the bioavailability of bilastine 3. All women of childbearing potential had to use an effective method of contraception during the study and 3 months thereafter.

After a washout period of 14–28 days, patients were assessed for critical temperature threshold (CTT) using the Temp Test 3.0®. Patients without a positive reaction at threshold testing discontinued their participation; these subjects were replaced. Microdialysis was performed to determine the levels of histamine, IL-6, IL-8 and TNF-α in the dermis before cold provocation and 0–20 and 20–60 min (histamine) and 2–3 h (cytokines) afterwards.

All eligible patients were then randomized to one of the two treatment groups, each containing the sequences placebo, bilastine 20 mg, 40 mg and 80 mg (Fig. 1). Patients were treated daily for 7 days followed by a 14-day washout period. At the end of each 7-day treatment phase, patients returned for the assessment of cold urticaria symptom development. Cold urticaria provocation and measurements of response were performed at all visits. Microdialysis was only performed before start of the study drug and after treatment with 20 mg and 80 mg bilastine.

### Medication, dosage and administration

Identical tablets containing either 20 mg bilastine or placebo were manufactured by FAES FARMA S.A. (Bilbao, Spain). Each morning for seven days before treatment visits, patients took four tablets, a mixture of bilastine 20 mg and placebo tablets, to achieve the required dose. To facilitate randomization, tablets were packaged in aluminium strips, each containing four tablets for each morning and labelled with patient randomization number and directions for use. Patients were instructed to take their medication at least 1 h after consumption of food and that fruit juice drinks should be avoided.

### Study readouts

#### Primary readout

Critical temperature threshold (CTT), defined as the highest temperature that produces a positive wheal response 11, was determined using a Temp Test® 3.0, a Peltier effect-based electronic device designed specifically for diagnosing and monitoring CCU symptoms 9. The temperature head of this device, which was placed directly on the volar surface of the forearm, consists of twelve elements, each 10 mm in diameter, arranged in two parallel rows. The device was set to deliver temperatures of 26, 24, 22, 20, 18, 16, 14, 12, 10, 8, 6 and 4°C (each ± 0.1°C) to the skin for a constant period of 5 min. A positive response is the development of a wheal, either confluent or nonconfluent, and itching within 5 min of removing the Temp Test 3.0®.

#### Secondary readouts

The severities of itching and burning of the cold-induced lesions were assessed 10 min after the end of cold provocation and recorded as: 0 absent, 1 mild, 2 moderate and 3 severe. The determination of pruritus and burning was performed at a single 4°C provocation site separate from that of the determination of CTT.

Microdialysis was used at visit V2 (baseline), visit V3 and visit V6 to determine the release of histamine and the generation of cytokines IL-6, IL-8 and TNF-α. Two linear cutaneous microdialysis membranes (3000 kDa molecular mass cut-off) were inserted into the volar surface of each forearm of the volunteers, under topical local anaesthesia (EMLA cream; Astra Pharmaceuticals, Kings Langley, UK) as previously described 15. Each membrane ran for a length of 20 mm, at a depth of approximately 0.7 mm. A 1-h period was allowed for recovery from local anaesthesia and trauma before the start of perfusion with Ringer's solution at a rate of 3 μl/min using a microinfusion pump. To assess baseline mediator levels, dialysate was collected from both probes for 30 min before cold provocation (4°C for 5 min) of the skin above one fibre. The other fibre served as unprovoked control. Dialysate was collected for the determination of histamine during two time periods, 0–20 and 20–60 min after the beginning of provocation. Further collections were made between 2 and 3 h for the determination of cytokines. Dialysate samples were collected in vials and stored at −80°C before analysis. Histamine was measured by Histareader™ (REFlab, Copenhagen, Denmark). Cytokines were measured by FlowCytomix™ Pro 2.4 (eBioscience, San Diego, CA, USA).

### Adverse events and follow-up

Patients were questioned at each visit about the occurrence of adverse events during the previous treatment period and washout phase, and a general physical examination, ECG and laboratory blood analyses were performed at the final visit. As this was a study involving a licensed and widely used H_1_-antihistamine, no formal follow-up was undertaken.

### Statistics

The results for critical temperature thresholds and pruritus are expressed as median (with 75% confidence limits) and the significance of differences is calculated using Wilcoxon's nonparametric test. The overall chi-square differences between treatments were calculated using Friedman's nonparametric test. The significance values for the numbers of individual patients responding or not responding to treatments were calculated using Fisher's exact test. The results for histamine and cytokines are expressed as mean ± standard error of mean (SEM) and the significance of differences is tested using Student's *t-*test for paired data.

## Results

### Critical temperature thresholds

#### Effectiveness of bilastine at standard dosing

The median CTT (with 75% confidence limits) for the placebo treatment was 18°C (8.5–22°C). In addition, only one patient was symptom free, that is, there was no evidence of a whealing response, at a provocation temperature of 4°C, the minimal testing temperature.

**Figure 2:**
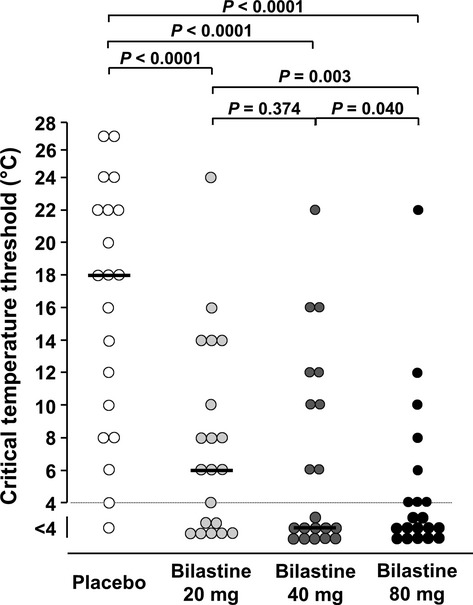
Critical temperature thresholds for the production of wheals following cold provocation. The horizontal lines indicate medians. The levels of significance values are for differences in the median critical temperature thresholds calculated using Wilcoxon's nonparametric test.

Following daily dosing for seven days with a standard dose of 20 mg bilastine, the median CTT value was 6°C (13 to <4°C), highly significantly different from the placebo treatment (*P* < 0.0001, Wilcoxon's *T* test) (Fig. 2, Table 1). Also, 7 (35%) patients became symptom free (*P* = 0.044 *vs* placebo, Fisher's exact test) at this dose.

**Table 1:** Individual response of critical temperature thresholds (CTT) to bilastine treatment

Patient number	Baseline	Placebo	Bilastine 20 mg	Bilastine 40 mg	Bilastine 80 mg
1	20	16	14	12	10
2	20	22	14	10	12
3	14	4	0	0	0
4	12	22	6	0	0
5	22	24	8	16	0
6	12	18	4	0	4
7	27	10	0	0	0
8	12	12	6	0	0
9	24	27	24	22	22
10	12	8	0	0	0
11	27	27	10	12	4
12	22	20	8	0	0
13	16	14	0	6	0
14	18	22	16	0	4
15	4	0	0	0	0
16	22	24	14	10	6
17	10	6	0	6	0
18	22	18	6	16	8
19	20	18	8	0	0
20	10	8	0	0	0
Median	19	18	6	0	0
75% Limit	22	22	13	11	5.5
25% Limit	12	8.5	0	0	0

#### Up-dosing with bilastine

The median CTT values following seven-day treatment with 40 mg and 80 mg bilastine were both below the minimal testing temperature of 4°C. The respective values of <4°C (11 to <4°C) and <4°C (5.5 to <4°C) were highly significantly (*P* < 0.0001, Wilcoxon's *T* test) different from those of placebo treatment (Fig. 2, Table 1). Furthermore, the median CTT with 80 mg bilastine was significantly lower than both that of 20 mg (*P* = 0.003) and 40 mg (*P* = 0.04), illustrating the benefit of up-dosing. In addition, 11 (55%) and 12 (60%) patients, respectively, became symptom free (*P* = 0.0012 and *P* = 0.0004 *vs* placebo, Fisher's exact test) on 40 and 80 mg bilastine. There were no significant differences between drug doses in the number of patients becoming symptom free.

The Friedman's chi-square test value of 41.836 indicates a highly significant (*P* < 0.0001, test) treatment effect of bilastine, taking all doses into consideration.

### Pruritus

**Figure 3:**
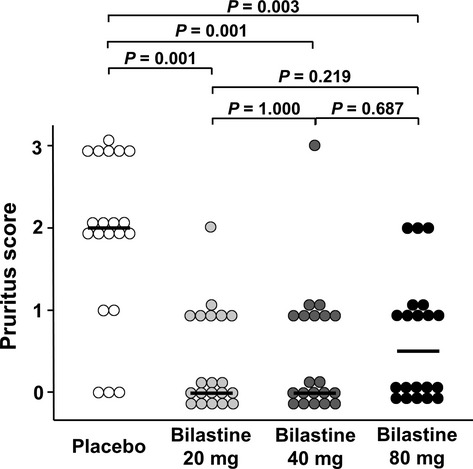
Pruritus scores following cold provocation. Pruritus was scored on a scale of 0–3. The horizontal lines indicate medians. The levels of significance values are for differences in the median scores for pruritus calculated using Wilcoxon's nonparametric test.

The median pruritus score (with 75% confidence limits) for the placebo treatment was 2.0 (1.25–3.0), and three patients reported no itch (Fig. 3). Following treatment with the standard bilastine dose of 20 mg daily, the median pruritus score was 0 (0–1) (*P* = 0.001 *vs* placebo, Wilcoxon's *T* test), with 13 of the 20 patients reporting no itch (*P* = 0.003 *vs* placebo, Fisher's exact test).

Following up-dosing with 40 mg and 80 mg bilastine, the median pruritus scores were 0 (0–1) and 0.5 (0–1). These were significantly different from placebo (*P* = 0.001 and *P* < 0.003, Wilcoxon's *T* test). In addition, 12 and 10 patients on 40 and 80 mg bilastine, respectively, reported no pruritus (*P* = 0.007 and *P* = 0.041 versus placebo, Fisher's exact test). There were no significant differences between doses.

The Friedman's chi-square test value of 26.650 indicates a highly significant (*P* < 0.0001, test) treatment effect of bilastine on pruritus.

Five patients in the placebo group reported a burning sensation following cold provocation, one at severity level 3 and two each at levels 2 and 1. Following administration of 20 mg bilastine, two patients reported burning, one at level 2 and one at level 1. With 40 mg bilastine, only one patient reported burning at level 1. With 80 mg bilastine, three patients reported burning, one at level 2 and two at level 1. These numbers were too low for meaningful statistical analysis.

### Levels of histamine and cytokines

#### Histamine

**Figure 4:**
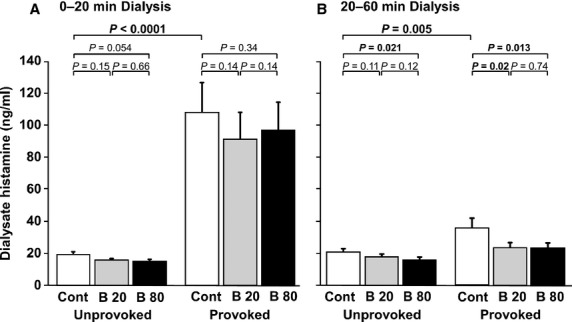
Dialysate levels of histamine. The white columns (Cont) are the samples obtained at visit 2 when no drug had been given. The grey and red-black columns (B20 and B80) are the samples obtained after administration of 20 and 80 mg of bilastine daily for 7 days. Each result is the mean ± SEM of results in 20 patients. The statistical differences were calculated using Student's *t*-test for paired data.

Histamine recovered by microdialysis from the unprovoked skin of untreated patients during 20 min was 19.2 ± 1.8 ng/ml (mean ± SEM). In the 0–20 min collection period following provocation, the concentration of histamine in the dialysate was 108.0 ± 18.1 ng/ml (Fig. 4A). This 5.6-fold increase was highly significant (*P* < 0.0001). The 5.7- and 6.4-fold increases of histamine in provoked skin compared with unprovoked skin in patients treated with bilastine at a standard 20 mg dose or up-dosed with 80 mg were not significantly different from those of untreated patients.

In untreated patients, the mean histamine level during the 20–60 min collection period in provoked skin of 35.7 ± 6.2 ng/ml was significantly (*P* = 0.005) higher than that in unprovoked skin of 20.9 ± 2.1 ng/ml (Fig. 4B). Following treatment with 20 or 80 mg bilastine, the mean histamine levels in provoked skin of 23.7 ± 3.2 and 23.5 ± 3.1 ng/ml were significantly (*P* = 0.02 and *P* = 0.013, respectively) lower than that in skin of untreated patients.

#### Cytokines

**Figure 5:**
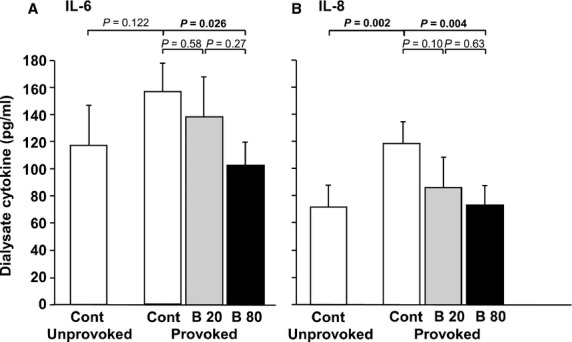
Dialysate levels of IL-6 and IL-8. The white columns (Cont) are the samples obtained at visit 2 when no drug had been given. The grey and black columns (B20 and B80) are the samples obtained after administration of 20 and 80 mg of bilastine daily for 7 days. All samples were collected between 2 and 3 h after the time of provocation. Each result is the mean ± SEM of results in 20 patients. The statistical differences were calculated using Student's *t*-test for paired data.

In untreated patients, cold provocation caused a small but not statistically significant increase in levels of IL-6 recovered by microdialysis (Fig. 5A). In contrast, cold provocation caused 60% increase in levels of IL-8 (*P* = 0.002) (Fig. 5B). Detectable baseline levels of TNF-α were found in only seven of the 20 patients and in only two of these did provocation lead to small increases.

Treatment of patients with a standard dose of 20 mg bilastine did not cause a significant reduction in either IL-6 or IL-8. However, up-dosing with 80 mg bilastine daily for 7 days caused a 34% (*P* = 0.026) reduction in IL-6 and a 37% (*P* = 0.004) reduction in IL-8 compared with when the patients were untreated.

### Adverse events

No severe adverse events or suspected unexpected serious adverse reactions occurred during this study. No volunteer withdrew from the study before its completion. A total of 17 adverse events (AEs) were reported. One patient reported sedation on one study day with 40 mg bilastine, one dizziness with 40 mg bilastine and two others headache alone (1 with 20 mg bilastine and 2 with 40 mg bilastine). However, these patients did not report these complaints consistently and not when taking the higher dose of 80 mg bilastine. Thus, these AEs are not considered to be drug-related. Other AEs, which were also probably not drug-related, included: exanthema (one patient, 40 mg bilastine), dyspnoea (one patient, placebo), bronchitis/laryngitis (three patients, 80 mg bilastine), upper respiratory tract infection (one patient, 20 mg bilastine), gastric pain (two patients, 20 mg bilastine; two patients, 40 mg bilastine; one patient, 80 mg bilastine) and constipation (one patient, 20 mg bilastine). There were no reported adverse responses after the end of the study.

## Discussion

This study showed that bilastine given daily for seven days before provocation is an effective H_1_-antihistamine in reducing the symptoms of cold contact urticaria, including wheal formation and pruritus. Increasing the dose of bilastine from 20 mg through 40 mg to 80 mg daily increased its effectiveness. Sedation was reported by only one patient with 40 mg bilastine. Histamine release was unaffected during the early phase of the response (0–20 min). However, levels of histamine collected during the 20- to 60-min period and IL-6 and IL-8 collected during the 2- to 3-hour period were significantly reduced, particularly when patients were treated with bilastine 80 mg.

The primary read-out in this study was assessment of critical temperature thresholds (CTT) which correlate the severity and activity of CCU 9, 14. When administered at the standard dose of 20 mg, bilastine was an effective inhibitor of CCU symptoms causing a reduction in the median CTT from 19°C to 6°C (68% reduction) and causing 35% of the patients to be symptom free with the lowest provocation temperature of 4°C. Increasing the dose of bilastine to 40 mg and then to 80 mg daily in line with the current EAACI/GA2LEN/EDF/WAO guidelines for the treatment for urticaria 1 significantly increased the effectiveness of the drug. With 80 mg bilastine, the median CTT was reduced to <4°C and 60% patients became symptom free.

In the first of three other studies using CTT as an output measure, desloratadine at 5 mg daily for one week reduced the mean CTT from 20.5°C to 15.2°C (26% reduction) with 7 of 30 (23%) patients becoming symptom free 11. Increasing the dose to 20 mg reduced the mean CTT to 11°C (47% reduction), with 50% of the patients becoming symptom free. In the second study, desloratadine at 5 mg daily for two weeks resulted in a reduction in mean CTT 22°C to 19°C (14% reduction), with no patients becoming symptom free 12. Increasing the dose to 20 mg further reduced the CTT to 12°C (45% reduction), with 5 of 15 (33%) patients becoming symptom free. In the third study, rupatadine at 20 mg daily for one week, twice the standard dose, reduced the median CTT from 18°C to 6°C (57% reduction), with 11 of 20 (52%) patients becoming symptom free 16.

Cold provocation produced a marked pruritic response in all but two patients during the period in which they were taking placebo. This response was significantly inhibited by bilastine at all doses. Because pruritus was assessed on a short four-point Likert scale, differences between doses were not apparent.

One outstanding feature of bilastine pharmacology is its interaction with transporter systems, including P-glycoprotein, which limit its penetration into the brain 5, 17. In addition, clinical objective psychomotor tests and subjective assessment of drowsiness indicate the absence of effects of bilastine on the central nervous system. These tests also show a lack of interaction of bilastine with lorazepam and alcohol 3, 18. The lack of sedation caused by bilastine, even with dose escalation, was highlighted in the present study by the fact that there was only one report of drowsiness by a single patient with 40 mg bilastine.

In untreated patients, there was a 5.6-fold increase in the concentration of histamine recovered by microdialysis during the first 20 minutes after cold provocation. This was not inhibited by bilastine at any dose. Although inhibition of histamine release from mast cells by H_1_-antihistamines, including bilastine, has been demonstrated often *in vitro,* the concentrations used are invariably higher than those usually reached during clinical therapy 19–23. Our observation that bilastine did not inhibit histamine release *in vivo* is consistent with previous reports using skin chambers or microdialysis that H_1_-antihistamines do not reduce histamine released in the skin following provocation with allergen or codeine 24–26.

In the microdialysis sample collected from untreated patients during the 20- to 60-min period, there was a small but significantly higher concentration of histamine in the provoked site compared with the nonprovoked site. Although the mechanism for this is not clear, it may be speculated that this is due to an influx and activation of basophils as is seen in late-phase responses to allergen 27–29 even though the cellular infiltrates in CCU are said to be ‘scanty’ due to the fleeting nature of these lesions 30.

The presence of an inflammatory response is supported by the observation of significant levels of IL-6 and IL-8, initially caused by the insertion of the microdialysis fibres 15. While allergen challenge significantly enhances the levels of both IL-6 and IL-8 15, cold provocation caused a small but significant rise in IL-8 and a small, but not statistically significant, rise in IL-6. This is consistent with the observation of a small up-regulation in CCU of cytokines observed by histology 31. Both IL-6 and IL-8 levels were significantly reduced by 80 mg but not 20 mg bilastine, again showing the advantage of up-dosing.

This study has shown that bilastine is an effective H_1_-antihistamine in reducing the symptoms of CCU when used in standard doses. Furthermore, up-dosing to 80 mg daily supports the urticaria treatment guidelines by showing excellent safety and no sedation while increasing its efficacy and anti-inflammatory properties.

## Abbreviations

CCU: cold contact urticaria
CTT: critical temperature threshold
EAACI: European Academy of Allergy and Clinical Immunology
EDF: European Dermatology Forum
GA^2^LEN: Global Allergy and Asthma European Network
IL-6: interleukin-6
IL-8: interleukin-8
PAF: platelet-activating factor
PGD_2_: prostaglandin D_2_
SEM: standard error of the mean
TNF-α: tumour necrosis factor-α
WAO: World Allergy Organization
